# Body mass index-associated molecular characteristics involved in tumor immune and metabolic pathways

**DOI:** 10.1186/s40170-020-00225-6

**Published:** 2020-09-25

**Authors:** Chao Hu, Xiong Chen, Chengyun Yao, Yu Liu, Haojun Xu, Guoren Zhou, Hongping Xia, Jinglin Xia

**Affiliations:** 1 Department of Pathology in the School of Basic Medical Sciences & The Affiliated Sir Run Run Hospital & State Key Laboratory of Reproductive Medicine & Key Laboratory of Antibody Technique of National Health Commission, Nanjing Medical University, Nanjing 211166, China; 2 The First Affiliated Hospital of Wenzhou Medical University, Wenzhou 325000, China; 3 The Affiliated Cancer Hospital of Nanjing Medical University & Jiangsu Cancer Hospital & Jiangsu Institute of Cancer Research, Nanjing 210009, China; 4 Department of Endocrinology, Sir Run Run Hospital, Nanjing Medical University, Nanjing 211166, China

**Keywords:** Body mass index, Pan-cancer analysis, Molecular characteristics, Tumor immune microenvironment, Metabolic pathways

## Abstract

**Background:**

Overweight or obesity has been evidenced as an important risk factor involved in the incidence, mortality, and therapy response of multiple malignancies. However, the differences between healthy and obesity tumor patients at the molecular and multi-omics levels remain unclear.

**Methods:**

Our study performed a comprehensive and multidimensional analysis in fourteen tumor types of The Cancer Genome Atlas (TCGA) and found body mass index (BMI)-related genes in multiple tumor types. Furthermore, we compared composite expression between normal, overweight, and obese patients of each immune cell subpopulation and metabolism gene subset. Statistical significance was calculated via the Kruskal-Wallis rank-sum test.

**Results:**

Our analysis revealed that BMI-related genes are enriched in multiple tumor-related biological pathways involved in intracellular signaling, immune response, and metabolism. We also found the different relationships between BMI and different immune cell infiltration and metabolic pathway activity. Importantly, we found that many clinically actionable genes were BMI-affect genes.

**Conclusion:**

Overall, our data indicated that BMI-associated molecular characteristics involved in tumor immune and metabolic pathways, which may highlight the clinical importance of considering BMI-associated molecular signatures in cancer precision medicine.

## Introduction

Excess body weight, including overweight and obesity, is defined as abnormal or excessive fat accumulation that increases the risk of many noncommunicable diseases [1]. A large number of studies reported that excess body weight is associated with the cancer burden. Epidemiological studies concluded that excess body weight increases the risk of 13 cancers with sufficient evidence, including the esophagus, gastric, colon and rectum, liver, gallbladder, pancreas, breast, corpus uteri, ovary, kidney, meningioma, thyroid, and multiple myeloma [2]. Besides, studies also reported that excess body weight is associated with other cancers, such as lung, prostate, and hematologic cancers [3–6]. Some studies indicate that obesity affects the treatment response of tumor patients. Obesity could promote anti-VEGF therapy resistance in breast cancer by producing inflammatory and angiogenic factors [7]. And obesity promoted cancer resistance to chemotherapy in breast, pancreatic, and prostate cancer [8–10]. Although obesity heightened immune dysfunction and tumor progression, greater anti-tumor efficacy and survival were found in obese patients treated with targeted therapy and immunotherapy [11, 12]. Previous studies have proposed various mechanisms to clarify the relationship between excess body weight and cancers. Chronic inflammation and metabolic abnormalities are the most studied [13–16]. Effects of adiposity-associated alterations of inflammation and microenvironment are thought to affect multiple cancers types, such as gastrointestinal, breast, liver, and pancreatic cancers [10, 17–20]. Insulin resistance and hyperinsulinemia can stimulate tumor cell proliferation and promote the growth of colorectal, pancreatic, liver, breast, and endometrial cancers [21, 22]. Adipose tissue affects the synthesis and bioavailability of sex hormones and mediates the association between excess body weight and hormone-related cancers [13, 16].

Body mass index (BMI), defined as body mass in kilograms divided by the square of height in meters (kg/m^2^), is the most widely used anthropometric measure to estimate overall body fatness and strongly correlated with adiposity [23]. Some studies have reported some BMI-related molecular patterns. For example, an obesity-associated cancer expression signature was defined in breast cancer [24]; gene microarray data revealed different signatures between obese and nonobese endometrial cancer patients [25]; PTEN loss resulted in PI3K pathway activation in nonobese patients, downregulation of β-CATENIN, and FOXO3A phosphorylation in obese patients in endometrial cancer [26], and DNA methylation pattern of excess body weight patients was changed in breast, colorectal, and kidney cancer [27–29]. However, these studies have been limited to individual genes, single-molecular data types, or single-cancer lineages.

The Cancer Genome Atlas (TCGA) has provided large-scale high-throughput molecular data with corresponding clinical data of multiple cancer types, which created an opportunity for researchers to systematically study the association between molecular data and BMI [30]. In this study, we performed a pan-cancer analysis to investigate the BMI-related comprehensive cancer molecular characteristics using these TCGA data.

## Materials and methods

### Analysis of patient clinical information

We obtained clinical information from TCGA-Clinical Data Resource (CDR) Outcome (https://gdc.cancer.gov/about-data/publications/pancanatlas) and Broad GDAC Firehose (http://gdac.broadinstitute.org/), the patients with both height and weight information were retained for subsequent analysis. BMI was calculated by body weight in kilograms divided by the square of height in meters (kg/m^2^). BMI differences between male and female patients were compared by the Wilcoxon rank-sum test. Patients can be classified into three categories (normal, BMI < 25; overweight, 25 ≤ BMI < 30; and obesity, 30 ≤ BMI) based on their BMI level, and survival difference between three groups was compared across all tumor types as well as individual tumor type with *p* value calculated via log-rank test using the *survival* package in R. And the clinical confounders such as age, gender, stage, and grade were controlled using multivariate COX analysis.

We obtained molecular subtypes information from *TCGAbiolinks* packages of Bioconductor. Furthermore, the BMI difference between different tumor subtypes was compared using the Kruskal-Wallis test.

### Analysis of mRNA expression and DNA methylation data

We obtained normalized gene mRNA expression and gene DNA methylation 450K data from the website of Firehose. The association between genes and BMI was calculated by propensity score weighting (PSW) method based on *PSW* package in R. The *p* value was adjusted by Benjamini & Hochberg method and genes with the adjusted *p* value less than 0.05 were considered as BMI-correlated genes; our method may miss few BMI-related genes after FDR control due to the limited number of tumor samples. Pathway enrichment analysis of selected BMI-correlated genes was applied using the *clusterProfiler* package. We also performed a Gene-Set-Enrichment analysis (GSEA) with the ranked gene according to the PSW and identified association between BMI and gene mRNA expression or methylation level by GSEA software 4.0 version and the significantly enriched pathways.

### Analysis of miRNA and protein expression data

We download the miRNA expression data from the Firehose database and the protein RPPA expression data from The Cancer Proteome Atlas (TCPA) database (https://www.tcpaportal.org/tcpa/index.html). The PSW method was used to identify BMI-associated genes and the adjusted *p* value less than 0.05 were considered as BMI-correlated miRNA or protein genes. To explore the potential functions of candidate miRNAs, we identified potential miRNA targets from the following experimentally supported miRNA-Gene interaction databases: miRTarbase, Tarbase, miRanda, miRDB, miRecords, and TargetScan. We then selected the candidate targets using two criteria: (1) the targets were in miRTarbase with strong evidence and (2) or in at least three of the other four databases. Pathway enrichment analysis was performed using these target genes.

### Analysis of somatic mutations and somatic copy-number alteration data

We obtained the somatic mutation (SNV) data from TCGA Pan-Cancer Atlas and significant somatic copy number alterations (SCNA) from Firehose. To prevent the potential effect caused by ultra-mutated samples, we filtered out the samples with over 1000 mutations in their exomes, while the non-silent mutations with over 5% mutation frequency in a patient cohort were retained for subsequent analysis because of their potential biological significance and detecting power in analysis. Next, we divided the patients into two groups, normal (BMI < 25) and excess weight (25 ≤ BM). The PSW method was used to compare SNV and SCNA versus normal patterns between normal and excess weight group patients. The BMI-related alterations were identified at adjusting *p* value (FDR) less than 0.1.

### Analysis of tumor-infiltrating lymphocytes and metabolism pathways

To study the BMI-related tumor immune microenvironment, we used the single-sample gene set enrichment analysis (ssGSEA) method to identify the tumor-infiltrating lymphocyte subpopulations described in a previous study [31]. We first obtained genes related to specific tumor-infiltrating lymphocytes from the study, which include 28 immune cell types. For each patient, genes were ranked according to their log-transformed expression. The association was represented by a normalized enrichment score (NES). An immune cell subpopulation was considered enriched in a patient when NES > 0 and FDR (*q* value) ≤ 0.1. For metabolism pathways, we obtained seven metabolic super-pathway gene sets that contain main human metabolic processes from the Reactome database. We applied an analysis strategy similar to the immune cell subpopulation analysis to analyze enriched metabolism pathways in each patient. Fisher’s exact test was used to compare enrichment patterns between normal and excess weight group patients. We compared composite expression between normal, overweight, and obese patients of each immune cell subpopulation and metabolism gene subset. Statistical significance was calculated via the Kruskal-Wallis rank-sum test.

### Analysis of BMI-related clinically actionable genes and drugs

The clinically actionable genes were defined as FDA-approved therapeutic targets and their corresponding predictor markers, which obtained from the TARGET database (http://www.broadinstitute.org/cancer/cga/target). We extracted the anti-cancer drugs and their target information from the DrugBank database.

## Results

### The BMI characteristics of TCGA samples

**Fig. 1:**
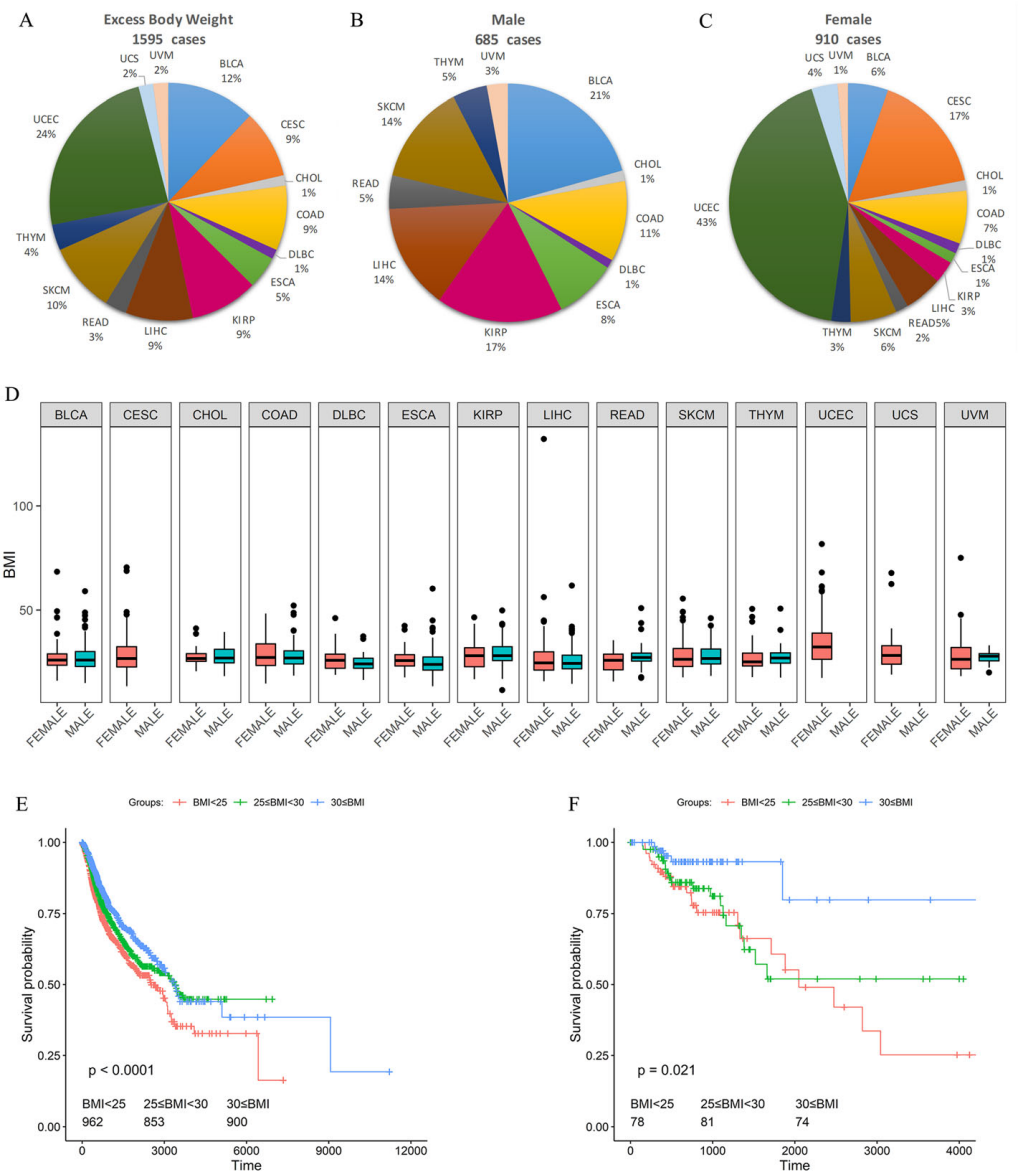
The BMI characteristics summary of TCGA patients. **a** The proportion of each tumor in all excess weight patients. **b** Proportion of each cancer in male excess weight patients. **c** Proportion of each tumor in female excess weight patients. **d** Comparison of the BMI difference between male and female patients for each tumor type, no significant difference found. **e** Survival difference between different BMI groups across all tumor patients. **f** Survival difference between diverse BMI group in COAD. The correspondence between full names and abbreviations is provided in Table S1

To characterize the specific molecular characteristics that are associated with body mass index (BMI), we selected 2715 patients across 14 tumor types that provided BMI information from The Cancer Genome Atlas (TCGA) project (Supplementary Table S1). Patients were divided into three categories according to the BMI (normal, BMI < 25; overweight, 25 ≤ BMI < 30; and obesity, 30 ≤ BMI). Eleven out of 14 tumors had more than half of excess body weight (25 ≤ BMI) patients, and endometrial carcinoma (UCEC) was the most significant contributor of excess body weight patients and also had the highest proportion across the 14 tumors (Fig. 1a). The total number of tumor cases with excess body weight was higher in females than in males (989/1471 in females vs. 768/1248 in men). UCEC was the largest cohort among female excess body weight patients, followed by cervical cancers (CESC) and colon adenocarcinoma (COAD). In contrast, the largest cohort among males was bladder carcinoma (BLCA), followed by kidney papillary cell carcinoma (KIRP) and hepatocellular carcinoma (LIHC) (Fig. 1b, c). No significant difference was observed between male and female patients’ BMI values (Fig. 1d).

To gain insight into the prognostic role of BMI, we analyzed the survival differences of the three BMI groups by Kaplan-Meier curves across all tumor patients. Patients with higher BMI corresponds to longer overall survival times across all patients (Fig. 1e). Survival analysis was also applied to each tumor separately, and only in COAD, we observed significant overall survival differences and obese patients had a better prognosis than normal and overweight patients (Fig. 1f). Furtherly, the obese was independently associated with patient prognosis after adjusted potential clinical factors with multivariate COX analysis (Figure S1).

### BMI-associated mRNA expression and DNA methylation characteristics

**Fig. 2:**
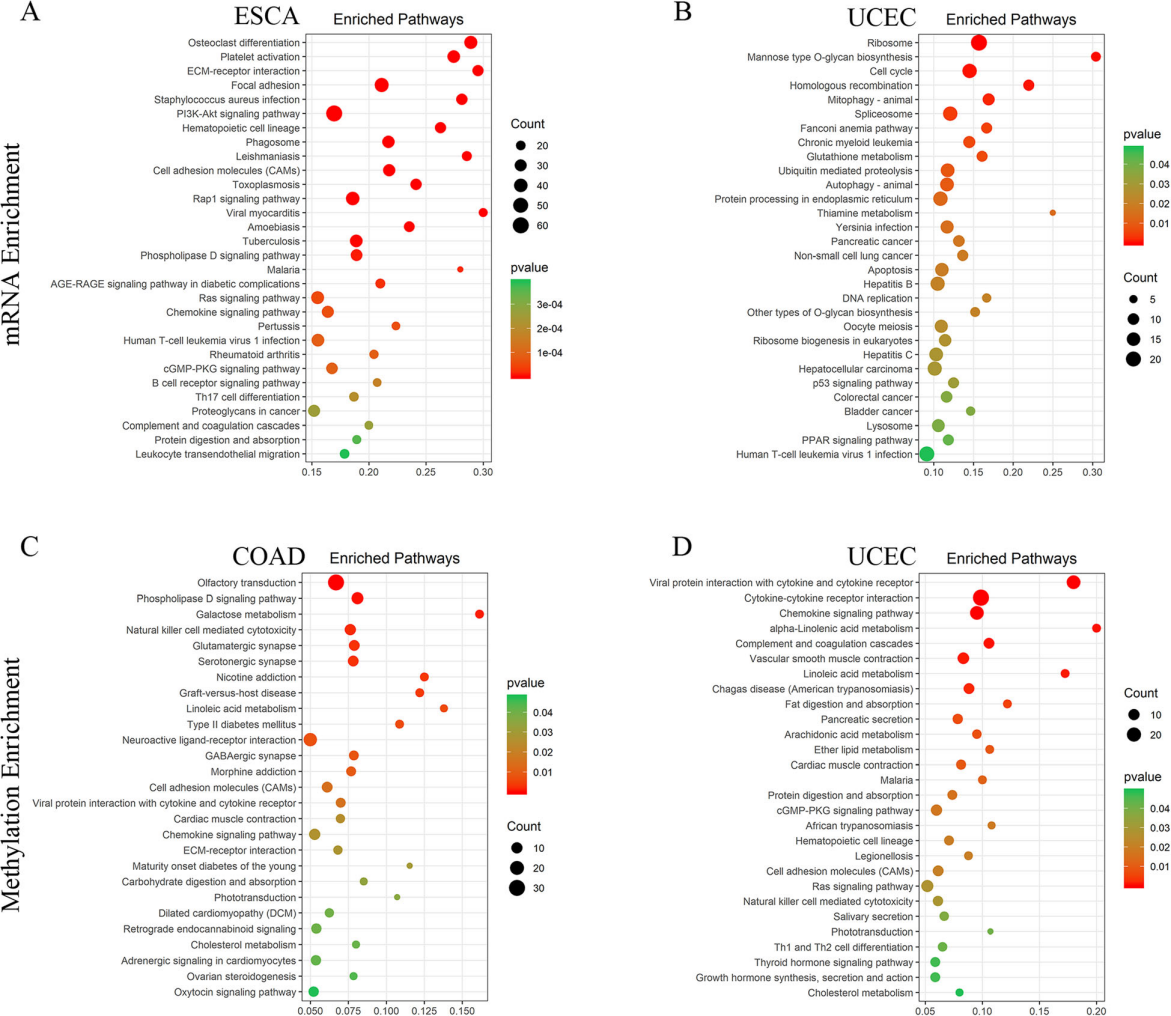
Expression signature of BMI-associated genes in three strong BMI-related tumors. The top-enriched KEGG pathways of BMI-correlated mRNA genes in **a** ESCA and **b** UCEC. The top BMI-correlated DNA methylation genes enriched KEGG pathways in **c** COAD and **d** UCEC

To characterize the BMI-associated gene mRNA expression and DNA methylation signatures, we performed a PSW analysis on 14 TCGA tumor types. For mRNA expression data, we detected BMI related genes in 8 tumors and only esophagus cancer (ESCA) and UCEC identified abundant genes associated with BMI, and for DNA methylation data, we detected BMI-related genes in 4 tumors and only COAD and UCEC identified abundant genes (Supplementary Table S2). To understand the functions of BMI-correlated genes in tumors, we performed a functional enrichment analysis and identified the affected KEGG pathways. In ESCA, BMI-associated mRNA genes were enriched in many important tumor pathways involved intracellular signaling, immune response, such as PI3K-Akt signaling, ECM-receptor interaction, and B cell receptor signaling pathways (Fig. 2a). A previous study report that BMI-associated differential genes in endometrial cancers were related to cilium/microtubule or cell cycle and DNA metabolic processes. It is similar to our result in UCEC that the BMI-related genes mostly enriched in cell cycle and metabolism-related pathways, we also identified BMI biases of CST3, ADAMTSL5, and ADAMTSL3 as mentioned related to cilium or microtubule (Fig. 2b). BMI-associated gene methylation enrichment showed that BMI-related genes were enriched in metabolic or metabolic diseases, natural killer cell cytotoxicity, and cell adhesion pathways in COAD (Fig. 2c). And in UCEC, the BMI-related gene methylations were associated with digestion and metabolism, immune cell activity, and signal transduction (Fig. 2d).

We also combined colon and rectal cancer as colorectal cancer (CORE) because of their clinical similarities. PSW analysis identified 17 mRNA and 1528 methylation genes associated with BMI in colorectal cancer. Pathway enrichment showed the genes were enriched in focal adhesion, cAMP signaling, and ECM-receptor interaction pathways (Figure S2A).

**Fig. 3:**
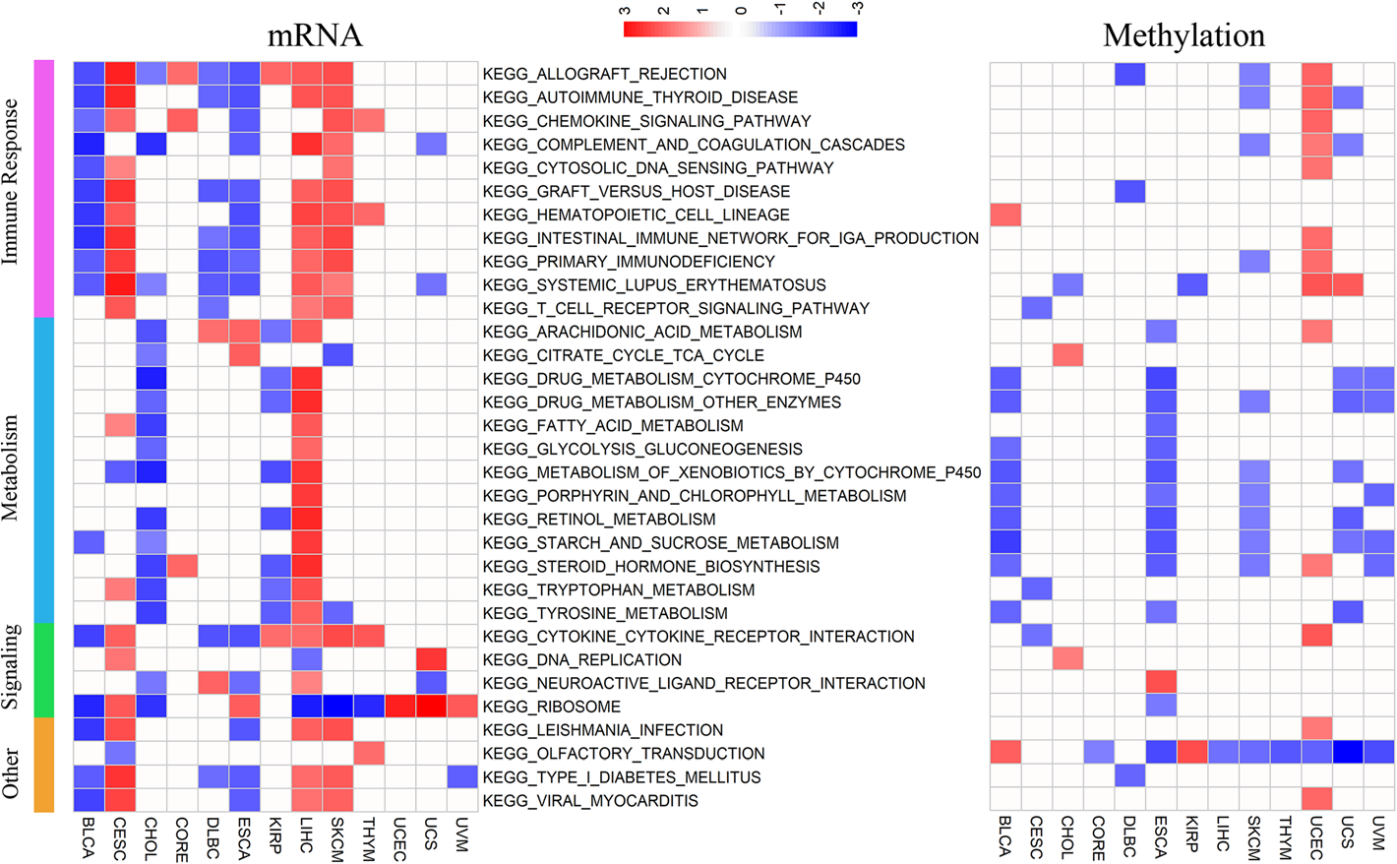
Expression signature of BMI-associated genes across TCGA cancers. The enriched pathways identified via GSEA of ranked mRNA and methylation genes, the color of each square represents NES values of significantly enriched pathways, and white squares indicate no statistical significance. The different colors of the bar on the left indicate the function group pathways belong to

We also performed a Gene-Set-Enrichment analysis (GSEA) given the gene ranks according to the association between BMI and gene expression or methylation level and identified the significant enriched biological pathways. Combining the gene expression and DNA methylation GSEA enrichment results, we found that the enriched pathways could be divided into four functional groups (immune response, metabolism, signal processing, and other pathways). The immune response group includes allograft rejection, complement and coagulation cascades, hematopoietic cell lineage, T cell receptor signaling, and primary immunodeficiency pathway. The pathways of metabolism group include citrate cycle, fatty acid metabolism, glycolysis and gluconeogenesis, retinol metabolism, steroid hormone biosynthesis, and tryptophan metabolism. The signal processing group are several genetic and environmental information pathways that are important in cancer, such as cytokine-cytokine receptor interaction, ribosome, and DNA replication (Fig. 3). ESCA and UCEC mRNA genes positively related to the ribosome, and ESCA mRNA negatively related to immune pathways while UCEC methylation genes are positively related to immune pathways, it is consistent with the reverse regulation of methylation on gene expression.

### BMI-associated miRNA and protein characteristics

**Fig. 4:**
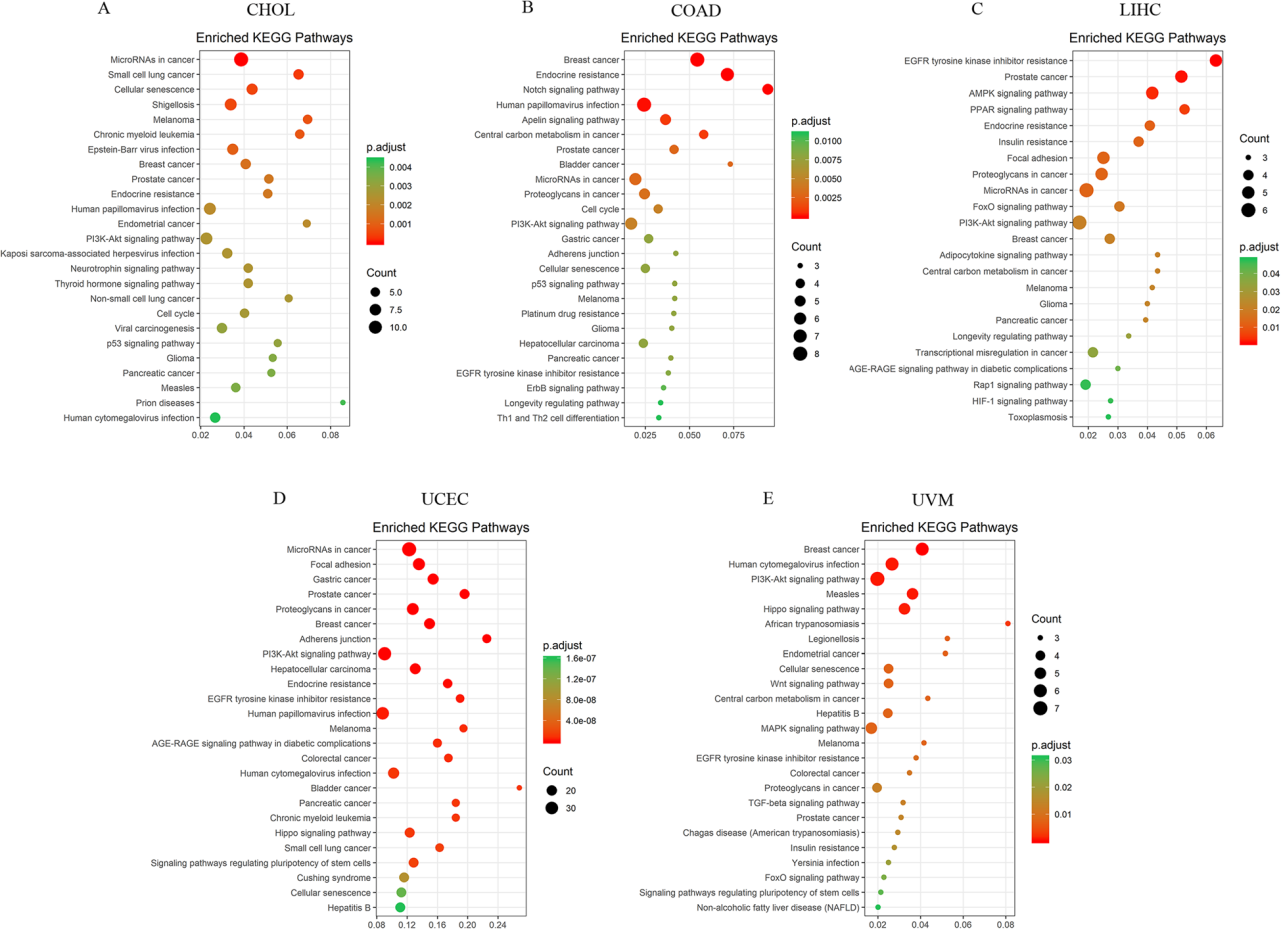
Enriched pathways of BMI-associated miRNA targets. The top-enriched KEGG pathways of BMI-related miRNA potential target genes in **a** CHOL, **b** COAD, **c** LIHC, **d** UCEC, and **e** UVM

To characterize the BMI-associated miRNA and protein expression, we also identified BMI-associated miRNA and protein via the PSW method. As for mature miRNA expression, we identified 1 in CESC, 3 in cholangiocarcinoma (CHOL), 9 in COAD, 2 in LIHC, 3 in rectum adenocarcinoma (READ), 17 in UCEC, and 23 in combined colorectal cancer (Supplementary Table S2). We obtained miRNA target information by integrating multiple miRNA-mRNA interaction databases based on 2 experimentally validated databases: miRTarBase and TarBase [32, 33] and 4 computationally predicted databases: miRanda, miRDB, miRecords, and TargetScan [34–37]. KEGG pathway enrichment analysis of miRNA target genes was applied, and we found that the function of miRNA-targeted pathways was similar and potential target genes of BMI-related miRNAs were significantly enriched in tumor-related signaling and cell proliferation pathways such as the PI3K-AKT signaling pathway (Fig. 4, Figure S2B).

For RPPA protein expression, we identified 6 BMI-related proteins (YWHAE, FN1, PRDX1, PRKCA, BAK1, and ANXA7) only in ESCA (Supplementary Table S2). Previously study report Mapk14-Ywhae signaling disorders in obese rats pancreas and increased YWHAE signaling promotes esophageal squamous carcinoma cell invasion [38, 39]. Dysregulated FN1 was identified in obese adipose tissue while high FN1 expression was associated with esophageal cancer [40, 41].

### BMI-associated somatic mutations and copy-number alteration characteristics

**Fig. 5:**
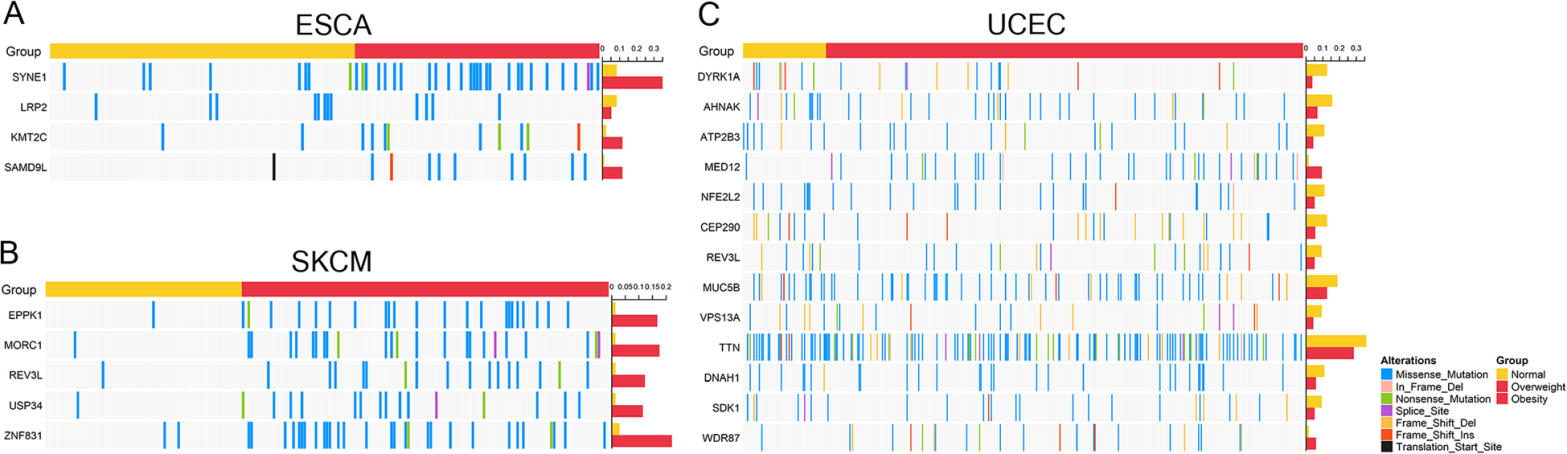
Signature of BMI-associated gene mutation across TCGA cancers. The mutation pattern of BMI-biased SNV between normal and excess weight patients in **a** ESCA, **b**S KCM, and **c** UCEC. The bar on the top is the BMI label of each patient, the bar on the right represents mutation frequencies of each BMI group, and different mutation types are shown in different colors

We next identified BMI-associated genomic level patterns. We focused on somatic mutations in each cancer type to identify BMI-associated mutation patterns using the PSW method. Four mutated genes were found in ESCA, 5 were found in SKCM, and 13 were found in UCEC (Supplementary Table S2). Among them, SYNE1, SAMD9L, and KMT2C were overweight-biased genes and LRP2 was normal-biased genes in ESCA, EPPK1, MORC1, REV3L, USP34, and ZNF831 were overweight-biased genes in SKCM, MED12, and WDR87 were overweight-biased while DYRK1A, AHNAK, ATP2B3, NFE2L2, CEP290, REV3L, MUC5B, VPS13A, TTN, DHAH1, and SDK1 were normal-biased genes in UCEC (Fig. 5).

**Fig. 6:**
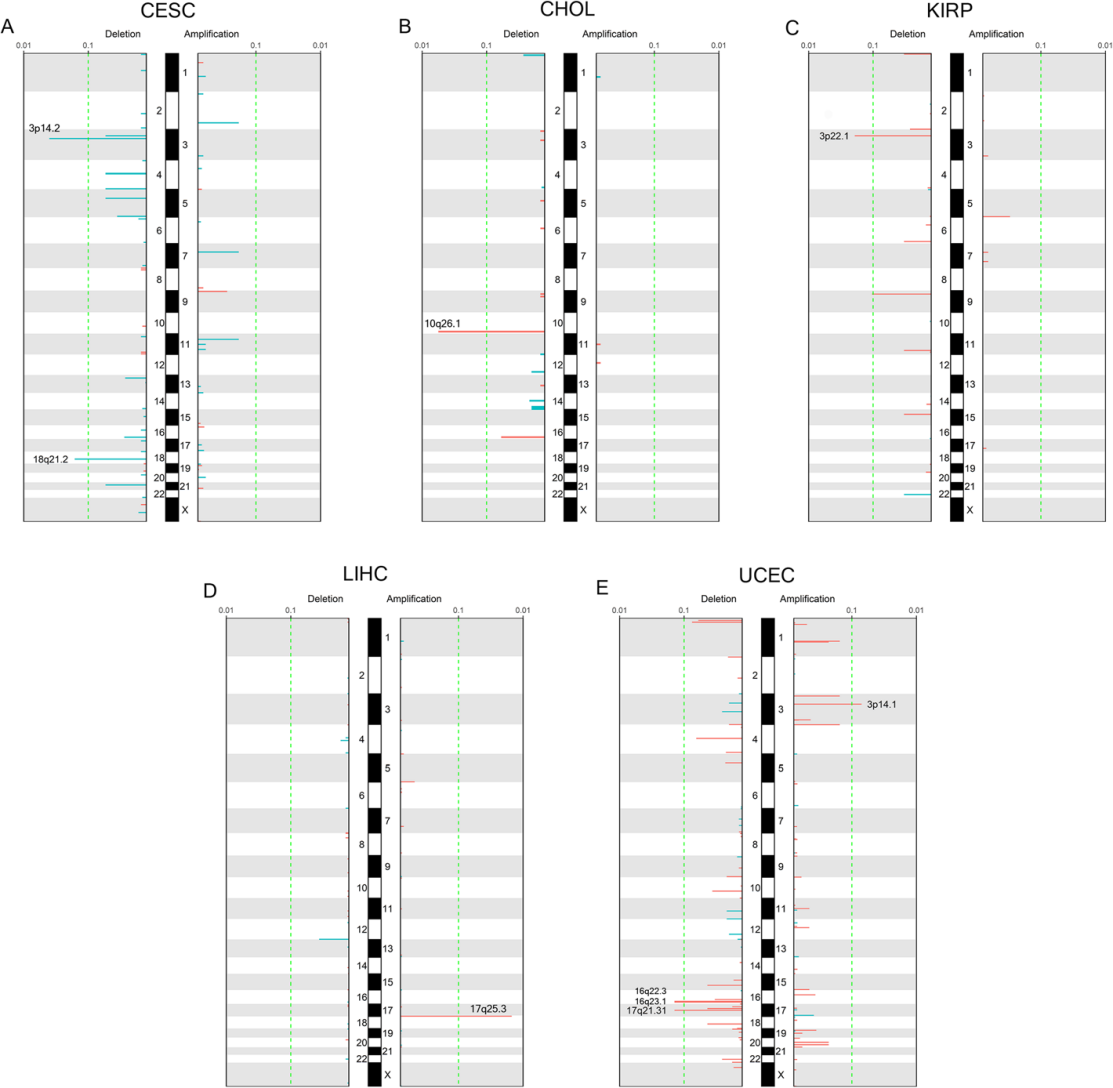
Signature of BMI-associated genome SCNV across TCGA cancers. The SCNV pattern of BMI-biased focal amplification/deletion in **a** CESC, **b** CHOL, **c** KIRP, **d** LIHC, and **e** UCEC. Each significant SCNA is annotated

To identify BMI-associated somatic copy-number alternations (SCNA), we analyzed the significant SCNA identified by GISTIC in each cancer type at focal level amplifications/deletions and identified BMI-associated SCNA in five tumor types. 3p14.2 and 18q21.2 deletion were normal-weight-biased in CESC; 10q26.1 deletion was excess-weight-biased in CHOL; 3p22.1 deletion was excess-weight-biased in KIRP; 17q25.3 amplification was excess-weight-biased in LIHC; 3p14.1 amplification, 16q22.3, 16q23.1, and 17q21.31 deletion were excess-weight-biased in UCEC and 5q23.3 deletion; and 7p21.3 amplification were excess-weight-biased in combined colorectal cancer at FDR = 0.1 (Fig. 6, Figure S2C, Supplementary Table S2). To understand the mechanism underlying BMI-related SCNA, we next examined enriched pathways of amplified or deleted genes and the result showed that copy number abnormal genes enriched in some critical biological process pathways, such as cell growth and differentiation pathways in CESC, protein translation process, and myeloid cell differentiation in KIRP, glucometabolic process, and epidermal growth factor receptor signaling in LIHC, cyclin-depend kinase activity in UCEC deletion genes (Figure S3).

### BMI associated with tumor molecular subtypes in LIHC and UCEC

The analysis of different molecular levels data showed that there were significant molecular differences in different BMI levels in particular tumor types, it is possible that obesity is associated with tumor molecular subtypes. So we compared the BMI difference between tumor subtypes and found that BMI distribution was significantly different only in LIHC and UCEC molecular subtypes (Figure S4A-B). The iCluster1 of the LIHC showed a low BMI feature [42]. The signature genes of the POLE subtype with the lowest BMI involved in cellular metabolism, while the CN-low subtype showed increased progesterone receptor expression [43]. It is interesting that the low BMI subtype has low CTNNB1 mutation frequency in LIHC, and the high BMI subtype has high CTNNB1 mutation frequency and the effects of the WNT-CTNNB1 pathway alterations on colorectal cancer outcome are modified by BMI and physical activity [44].

### The association between BMI and tumor immune microenvironment

**Fig. 7:**
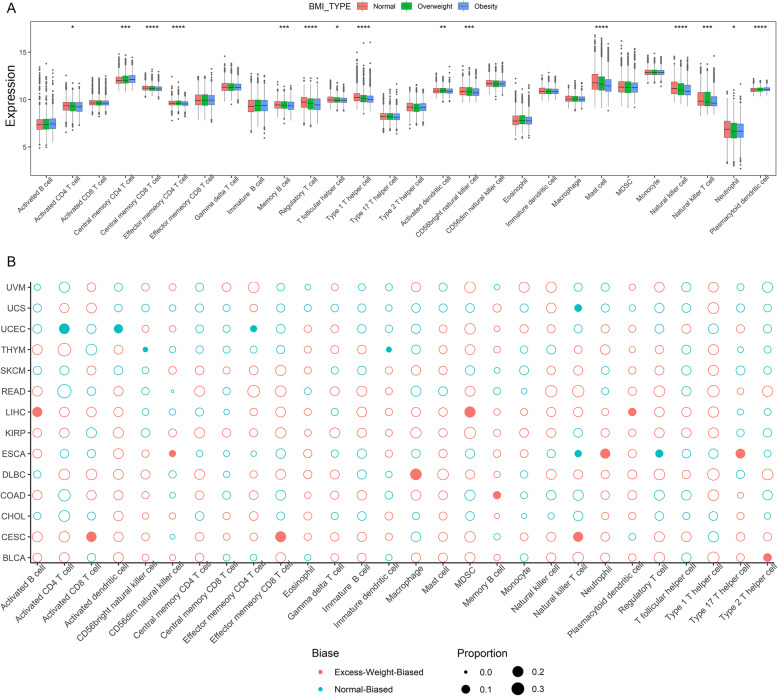
The chariotries of immune cell subpopulations in different BMI groups. **a** The composite expression of each immune cell gene subset in normal, overweight, and obesity patients across all tumor types, with statistical significance calculated by the Kruskal-Wallis rank-sum test. *p value < 0.05, **p value < 0.01, ***p value < 0.001, and ****p value < 0.0001. **b** The proportion of immune cell-enriched patients in excess weight group, each point represents an immune cell type in each tumor type with the size corresponding to the percent of enriched patients. Filled points corresponding to significant enrichment differences of immune cells between normal and excess weight patients

To study the association between BMI and tumor immune microenvironment, we first obtained genes related to specific tumor-infiltrating lymphocytes described in the previous study, which include 28 immune cell types [31]. Considering the effect of tumor purity on immune cell infiltration, we compared the purity between different BMI groups and no statistically significant difference was observed (Figure S4B). We then compared the composite expression of each gene subset, which represents distinct immune cell subpopulations, in different BMI groups and estimated tumor-infiltrating lymphocytes from TCGA expression data by both single-sample gene set enrichment analysis (ssGSEA) and CIBERSORT approaches [45, 46]. Fifteen of 28 immune cell types showed significantly different expressions in different BMI groups across all cancer types. Among them, only two immune cell subpopulations (central memory CD4 T cell and plasmacytoid dendritic cell) showed a positive correlation with BMI, while the others were negatively correlated (Fig. 7a). For individual tumor types, we also observed multiple immune cell subpopulation expression differences in CESC, ESCA, LIHC, and UCEC (Figure S5).

Using the ssGSEA strategy, we estimated the 28 immune cell subpopulations in the tumor microenvironment of individual patients. For each immune cell subpopulation of each tumor type, we compared the enrichment and survival difference between normal weight and excess weight patients. We found that the enrichment status of more than one immune cell subpopulation is different between normal weight and excess weight patients in CESC, ESCA, LIHC, THYM, and UCEC (Fig. 7b).

The fractions of 22 immune cell subpopulations identified by CIBERSORT were obtained from a previous study [47]. We then analyzed the association between BMI and tumor immune microenvironment. BMI was positively correlated with activated NK cells and CD8 T cells in CESC, activated mast cell, monocytes, resting memory CD4 T cells in ESCA, neutrophils in KIRP, resting dendritic cells and T regulatory cells in UCEC, follicular helper T Cells in UVM, but BMI was negatively correlated with M1 macrophages and M2 macrophages in ESCA, follicular helper T Cells in UCEC (Figure S6).

The immune cell types assessed by ssGSEA and CIBERSORT were not completely consistent, but we also found that the CD8 T cell in CESC was positively correlated with BMI in both methods. The above data suggested that BMI may play an important role in tumor immune microenvironment and may affect the different responses of cancer immunotherapy in the clinic.

### The association of BMI with different tumor metabolic pathways

**Fig. 8:**
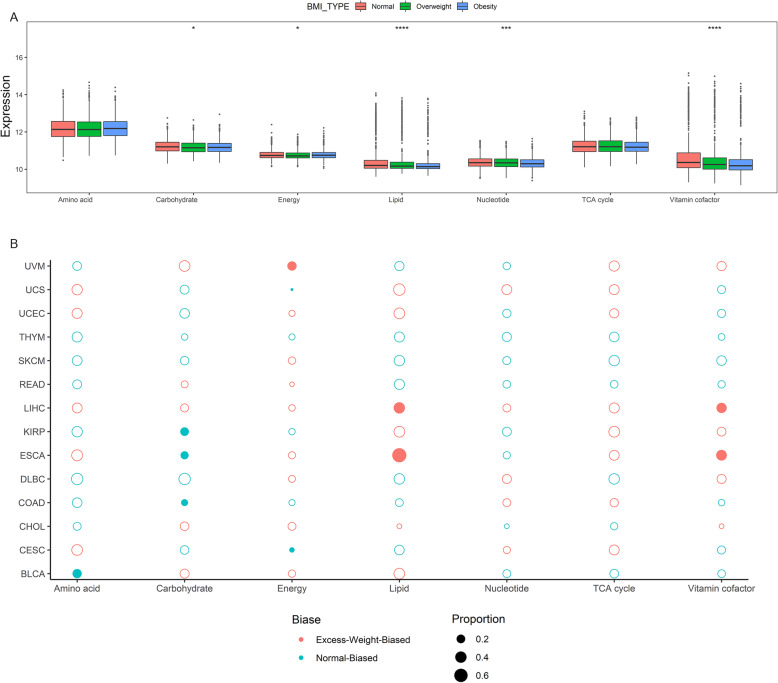
The chariotries of metabolism pathways in different BMI groups. **a** The composite expression of each metabolism pathway genes in normal, overweight, and obesity patients across all tumor types, with statistical significance calculated by the Kruskal-Wallis rank-sum test. *p value < 0.05, **p value < 0.01, ***p value < 0.001, and ****p value < 0.0001. **b** The proportion of metabolic pathways enriched patients in excess weight group. Each point represents a metabolic pathway in each tumor type with the size corresponding to the percent of enriched patients. Filled points corresponding to significant enrichment difference of metabolic pathway between normal and excess weight patients

To gain a penetrating view of metabolic heterogeneity in different BMI groups, we obtained the gene sets of seven metabolic super-pathways based on the Reactome database [48]. Seven metabolic pathways include amino acid metabolism pathway with 348 genes, carbohydrate metabolism pathway with 286 genes, energy metabolism pathway with 110 genes, lipid metabolism pathway with 766 genes, nucleotide metabolism pathway with 90 genes, tricarboxylic acid (TCA) cycle pathway with 148 genes, and vitamin-cofactor metabolism pathways with 168 genes. We compared the composite expression of each gene subset among different BMI group samples and found differential expression in carbohydrate, energy, lipid, nucleotide, and vitamin-cofactor metabolism pathways (Fig. 8a). For individual tumor types, we found differential expression metabolism pathways in BLCA, COAD, ESCA, KIRP, READ, and UCEC (Figure S7).

We also estimated this seven metabolism pathway enrichment of individual patients using the ssGSEA method. In 8 cancer types, we found at least one differential enrichment metabolic pathway between normal and excess weight groups (Fig. 8b).

### BMI-related molecular signatures in clinically actionable genes

**Fig. 9:**
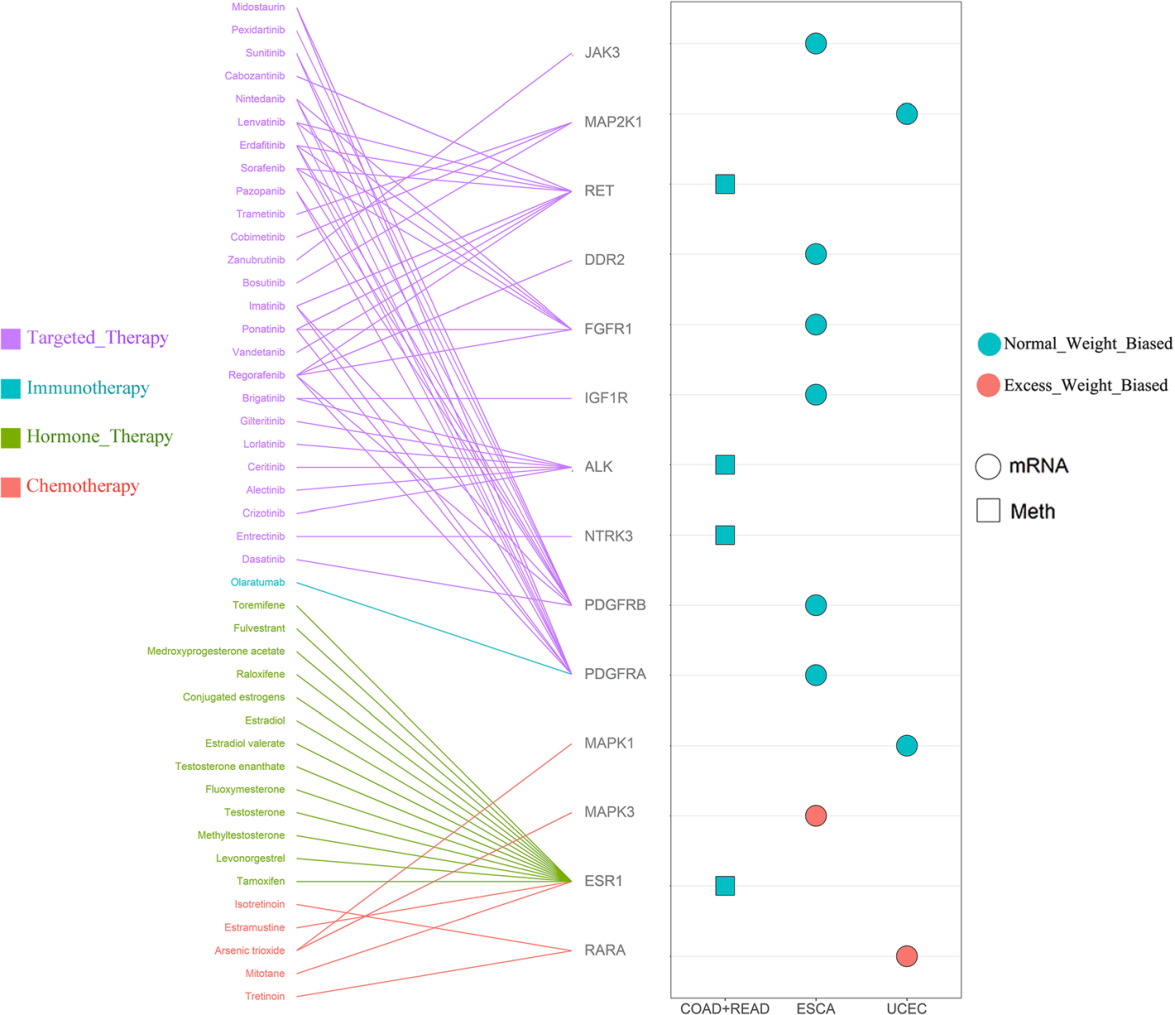
Signature of BMI-related clinically actionable genes and FDA-approved drugs. The target relationship between FDA-approved anti-cancer drugs and corresponding clinically actionable genes (left) and the observed BMI-associated clinically actionable genes in each tumor type (right). A different color of symbols indicated different shapes of symbols indicated BMI groups and molecular models

To investigate the clinical implications BMI-related molecular signatures, we focus on a set of clinically actionable genes, which are the targets of FDA-approved anti-cancer drugs [49]. We selected our BMI-related clinically actionable genes following two criteria: (1) the target genes were contained in BMI-related genes and (2) the drugs of the targets were recorded as being able to be used for cancer treatment. We found 44 drugs target in 14 clinically actionable genes across different tumor types (Fig. 9). These 44 drugs can be categorized into four groups: chemotherapy, hormone therapy, immunotherapy, and targeted therapy. Previous studies reported PDGFRs as adipogenesis negative regulators and were associated with tumor stroma and survival [50, 51], and their biases in different BMI groups may suggest treatment response differences with olaratumab or dasatinib. Chronic myeloid leukemia observed significant obesity gain after imatinib treatment [52]. These results highlight the clinical importance of considering BMI-associated molecular signatures in precision medicine.

## Discussion

Although the effect of overweight or obesity on tumor incidence, prognosis, and treatment responses has been reported in many kinds of literature, the molecular basis has remained unclear. Our study applied a comprehensive pan-cancer analysis that aimed to found BMI-related molecular differences across different cancer types and systematically catalogs the molecular signatures related to the BMI effect from genome to transcriptome to proteome level. We found colorectal, esophageal, and endometrial cancer were strong BMI-effect based on abundant molecular signatures in multidimensional data. Some of the BMI-correlated genes identified in this study have also been reported in different tumors by previous studies. For example, we identified BMI-correlated genes AKAP5 and JMJD5 in CESC, while it was reported that knockdown AKAP5 inhibited ERK1/2 activity and downregulation JMJD5 suppresses oral squamous cell carcinoma metastasis and induces apoptosis via p53/NF-κB pathway [53, 54].

Enrichment analysis shows that BMI-related genes are enriched in some important tumorigenesis and cancer development pathways, such as cell adhesion, cell proliferation, intracellular signaling, and specific tumor pathways. BMI-related genes enriched in p53 signaling pathways in UCEC, as it was reported that diet-induced obesity synergized with p53 mutation promoted hepatocarcinogenesis in zebrafish [55]. TP53 positivity was associated with shorter cancer-specific survival in nonobese patients, and breast and prostate cancer cells with mutant p53 increased oncogenic insulin effects [56, 57]. Obese tumors patients with PI3K pathway mutant had a trend toward favorable outcomes [58]. GSEA analysis result indicated that signaling processing, metabolism, and immune response are the main BMI-affected pathways class in tumors. Previously, a study reported IGF system played an essential role in esophageal cancer malignant progression and association with visceral obesity [59]. Our study found IGF1R and some other IGF family genes significantly correlated with BMI of esophageal cancer patients, indicating the IGF pathway might be played a differential role in tumor progression between obesity and normal body weight esophageal cancer patients. Global DNA methylation differential analysis of esophageal tumor showed differential methylation genes between different BMI groups involved in cell adhesion molecules, Wnt signaling, and growth hormone response, while our result showed a significant difference in cell adhesion, PI3K-AKT signaling, and Ras signaling pathways between the different BMI level patients [60]. Obesity is a risk factor strongly associated with endometrial cancer. Our study reveals that BMI-related genes were involved in the cell cycle, cell junction pathways, and significantly correlated with hormone receptor PGR in endometrial carcinoma. Previously researches reported the functions of adipose tissue in hormone production, inflammatory responses, and cellular proliferation pathways, while adipokines regulate cellular communication via gap junction loci [61, 62]. And the hormone receptor ER and PR were significantly low express after bariatric surgery in endometrial and reduced cancer risk [63].

Obesity is a risk factor strongly related to tumor immune, inflammation, and metabolism. Previously, research demonstrated the association between obesity and immune aging, tumor progression, and PD-1-mediated T cell dysfunction and increased efficacy of PD-1/PD-L1 blockade in tumors [11]. Obesity-associated inflammation in cancer associated with multiple immune cell subsets such as natural killer cells, macrophages, and T cells [64]. Moreover, the presence of a B cell is related to good immunotherapy response in sarcoma and melanoma [65, 66]. Our study found the enrichment difference in multiple types of immune cells, such as T cell, the natural killer cell in cervical cancer, B cell in liver cancer, and natural killer T cell in uterine carcinosarcoma. This may be relevant to the immunotherapy response difference in obesity patients.

## Conclusions

Our results provide a valuable starting point from which BMI effects should be explicitly considered in future clinical investigations and oncotherapy. Since TCGA clinical information may not be complete and rigorously annotated, approximately only 2700 patients recorded their BMI information across 14 cancer types. Future studies about this topic should conduct analysis larger cancer patient cohort and more tumor types, and the effects of BMI caused normal tissue to alter, and other potential confounding factors should be taken into consideration, as well as the efforts of assessing the clinical utility of the identified BMI-associated signatures.

## Supplementary information

**Additional file 1: Figure S1.** The association between BMI and survival by Multivariate COX analysis. **Figure S2.** Methylation, miRNA and SCNV characteristic in CORE. **Figure S3.** Pathway enrichment of BMI-biased SCNV signature. **Figure S4.** The association between BMI and tumor subtype or purity. **Figure S5.** The chariotries of immune cell subpopulations in different BMI groups of each tumor type. **Figure S6.** Chariotries of CIBERSORT identified immune cell subpopulations in different BMI groups of each tumor type. **Figure S7.** The chariotries of metabolism pathways in different BMI groups of each tumor type.

**Additional file 2: Table S1.** BMI characteristics of TCGA samples.

**Additional file 3.** The BMI-related genes.

## Data Availability

The datasets used and/or analyzed during the current study are available from the corresponding author on reasonable request.

## Abbreviations

TCGA: The Cancer Genome Atlas
BMI: Body mass index (BMI)
CDR: Clinical Data Resource
MAPK: Mitogen-activated protein kinases
SCNA: Somatic copy-number alternations
ssGSEA: Single-sample gene set enrichment analysis
NES: Normalized enrichment score
