# Supplementary figures and images for: Body mass index-associated molecular characteristics involved in tumor immune and metabolic pathways
Source: Cancer Metab. 2020 Sep 25;8:21. doi: 10.1186/s40170-020-00225-6 (PMC7517824; doi:10.1186/s40170-020-00225-6)

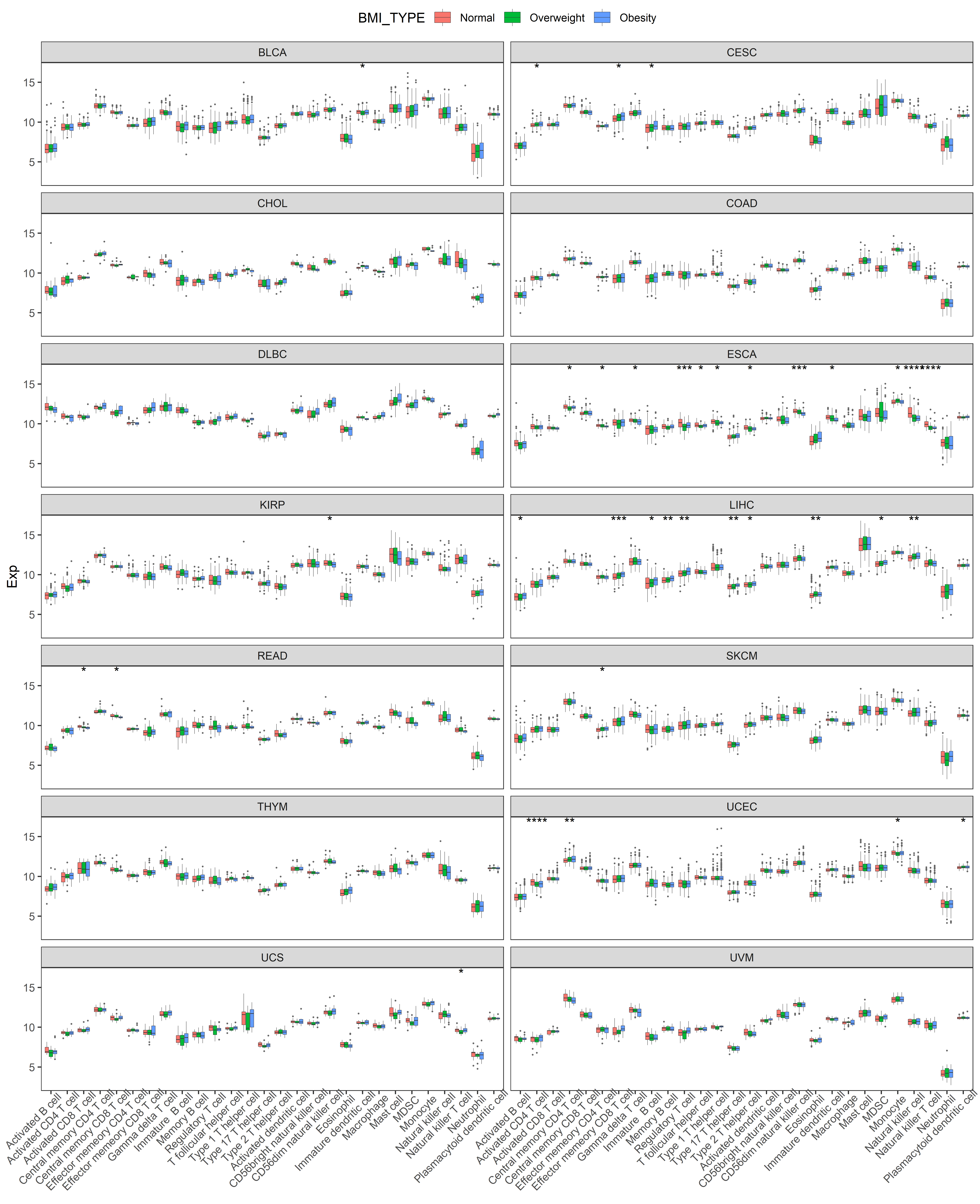

Supplement: Supplementary file 1 — Additional file 1: Figure S1. The association between BMI and survival by Multivariate COX analysis. Figure S2. Methylation, miRNA and SCNV characteristic in CORE. Figure S3. Pathway enrichment of BMI-biased SCNV signature. Figure S4. The association between BMI and tumor subtype or purity. Figure S5. The chariotries of immune cell subpopulations in different BMI groups of each tumor type. Figure S6. Chariotries of CIBERSORT identified immune cell subpopulations in different BMI groups of each tumor type. Figure S7. The chariotries of metabolism pathways in different BMI groups of each tumor type. [file 40170_2020_225_MOESM1_ESM.zip › Figure-S5.tif]

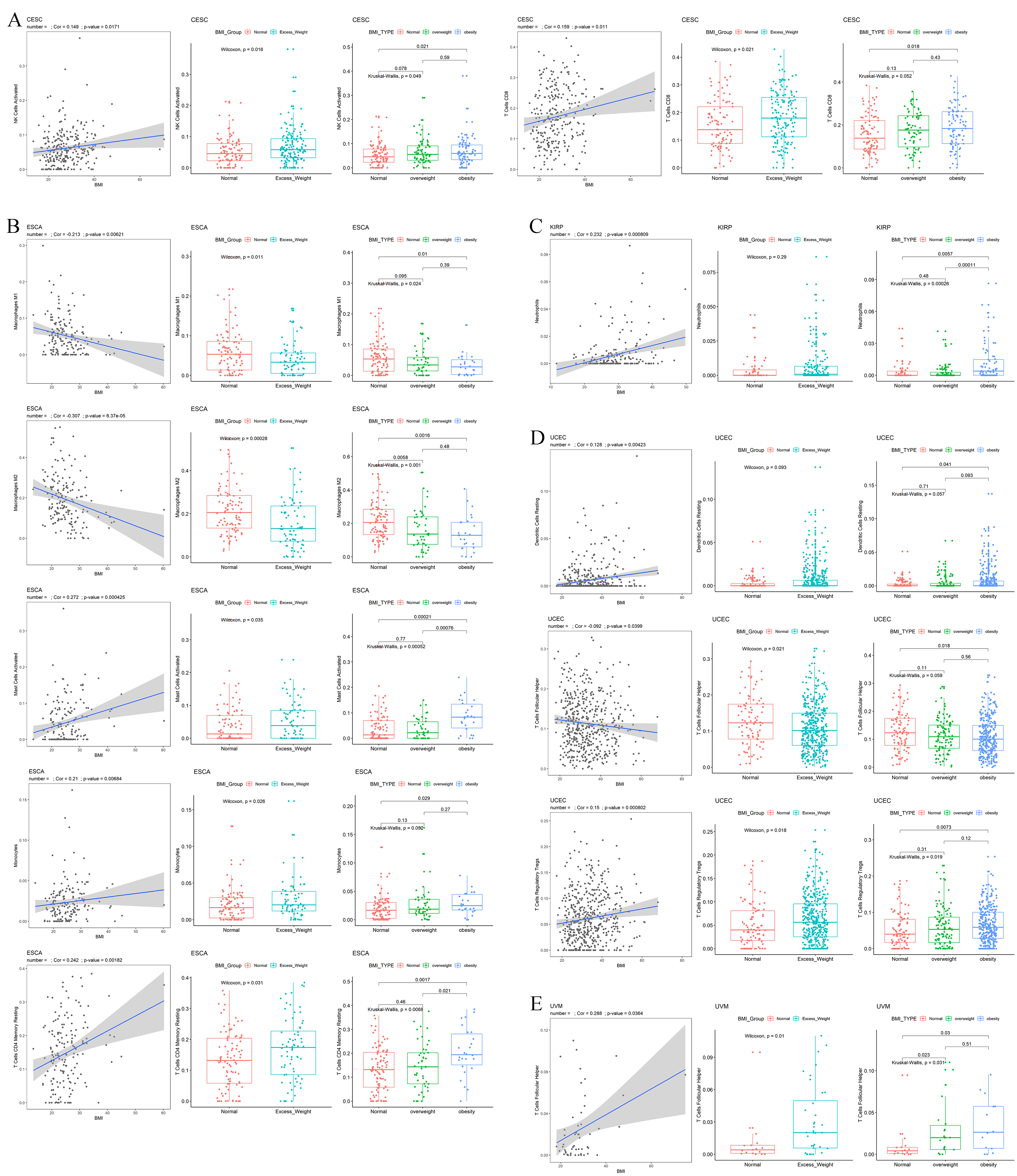

Supplement: Supplementary file 1 — Additional file 1: Figure S1. The association between BMI and survival by Multivariate COX analysis. Figure S2. Methylation, miRNA and SCNV characteristic in CORE. Figure S3. Pathway enrichment of BMI-biased SCNV signature. Figure S4. The association between BMI and tumor subtype or purity. Figure S5. The chariotries of immune cell subpopulations in different BMI groups of each tumor type. Figure S6. Chariotries of CIBERSORT identified immune cell subpopulations in different BMI groups of each tumor type. Figure S7. The chariotries of metabolism pathways in different BMI groups of each tumor type. [file 40170_2020_225_MOESM1_ESM.zip › Figure-S6.tif]

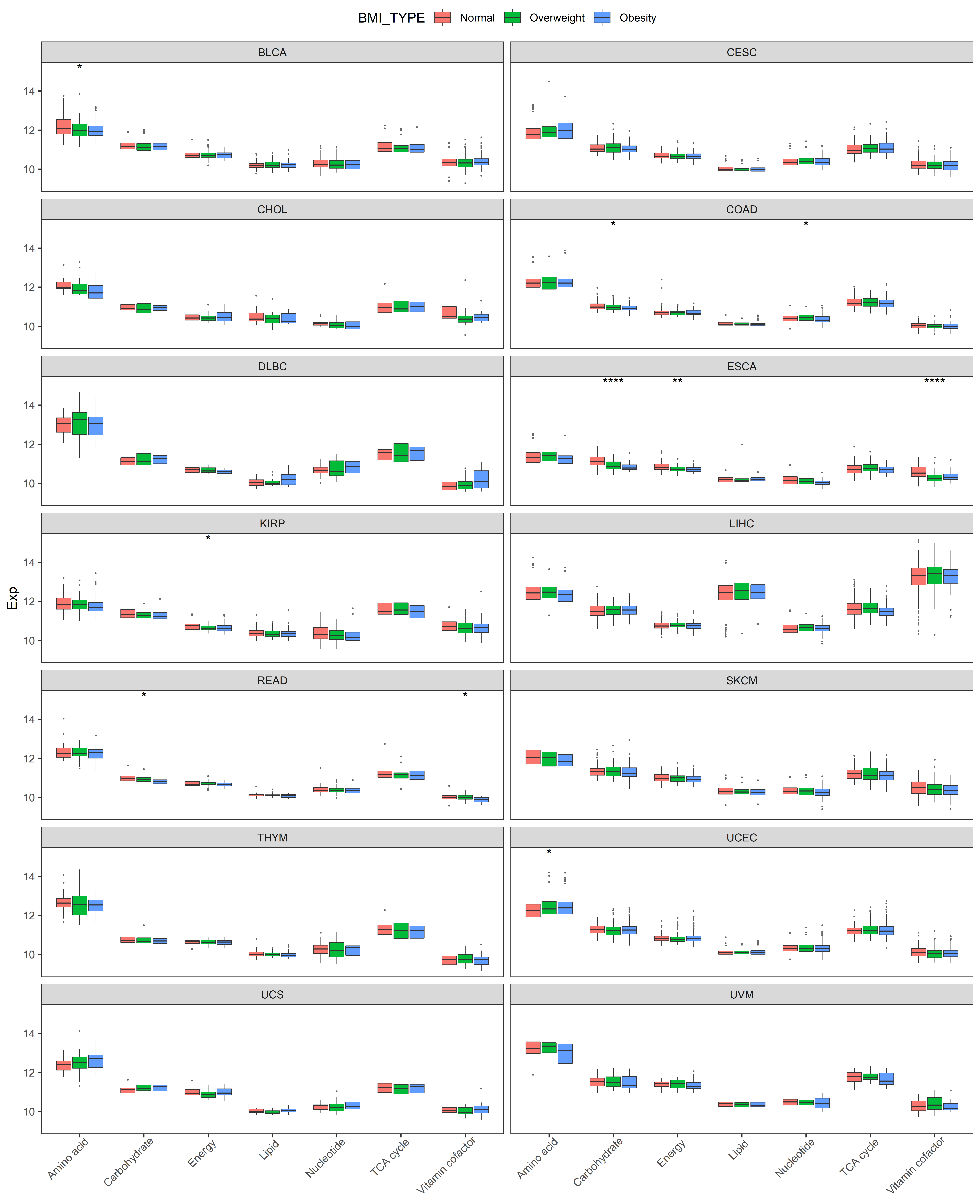

Supplement: Supplementary file 1 — Additional file 1: Figure S1. The association between BMI and survival by Multivariate COX analysis. Figure S2. Methylation, miRNA and SCNV characteristic in CORE. Figure S3. Pathway enrichment of BMI-biased SCNV signature. Figure S4. The association between BMI and tumor subtype or purity. Figure S5. The chariotries of immune cell subpopulations in different BMI groups of each tumor type. Figure S6. Chariotries of CIBERSORT identified immune cell subpopulations in different BMI groups of each tumor type. Figure S7. The chariotries of metabolism pathways in different BMI groups of each tumor type. [file 40170_2020_225_MOESM1_ESM.zip › Figure-S7.tif]
